# Role of Advanced Diagnostic Aids in the Detection of Potentially Malignant Disorders and Oral Cancer at an Early Stage

**DOI:** 10.7759/cureus.34113

**Published:** 2023-01-23

**Authors:** Nishath Sayed Abdul

**Affiliations:** 1 Department of Oral Maxillofacial Surgery (OMFS) and Diagnosis Sciences, College of Dentistry, Riyadh Elm University, Riyadh, SAU

**Keywords:** survival rate, histopathological evaluation, oral cancer, oral pathology, diagnostic aids

## Abstract

One of the most prevalent malignancies diagnosed today is cancer of the mouth or oral cancer. Compared to systemic malignancies like lung cancer, colon cancer, etc., oral cancer tends to get less attention from the general public. However, these lesions may be lethal if not treated, even if diagnosed early. Early diagnosis improves the prognosis for successful therapy. Delayed diagnosis is hypothesized to be a pivotal contributor to the dismal oral cancer survival rate over five years. The current standard of care for diagnosis and detection is based on clinical evaluation, the histological study of biopsy material, and genetic methods. There have been several advancements in the diagnostic technologies available to detect oral cancer at the initial phase. This study aims to dissect the cutting-edge methods for detecting oral cancer in its earliest stages.

## Introduction and background

It is widely considered that the diagnostic lag in oral cancer cases is attributable to a lack of public knowledge about the disease's signs, symptoms, and risk factors [1]. They are notoriously hard to diagnose with a standard clinical examination or checkup. Sir Edward Home's 1830 paper was the first to describe human malignancies at the microscopic level, followed by Johannes Muller's 1838 explanation [2]. It was not until the 1800s that histotechnology became prevalent and wax embedding of specimens became the standard method of preserving biological material for study. In the twenty-first century, frozen sections became standard practice in American hospitals. Using microscopic analysis of tissues and cells, the French botanist Francois-Vincent Raspail allegedly discovered the chemical process first, as reported by Pearse [3].

Over the last several decades, pathology has flourished, significantly contributing to our knowledge of the causes and consequences of genetic abnormalities and the identification of many forms of malignant tumors. Clinical applications of molecular methods have been increasing for the last two decades. The field of pathology has recently seen many advancements, such as brush cytology, telescope, confocal microscopy, tumor markers, microarray, etc. Robotics, humanoid technology, lab-on-a-chip gadgets, nanodevices, and patient, intelligent implants are all technologies that will soon make it feasible for laboratories to explore fascinating new territory. The lack of sophisticated tools for manipulating and sectioning human tissues slowed the development of surgical pathology. Until the 1800s, embedding specimens in wax was a relatively uncommon practice, and histotechnology was in its infancy. By the start of the twentieth century, frozen sections were routinely used in American hospitals and were widely regarded as safe [4]. This research proposal has been registered at the research center of Riyadh Elm University with registration number FRP/2022/478/863.

## Review

Potentially malignant disorders are difficult to diagnose using the conventional clinical examination. The diagnosis of these conditions is predicated primarily on a microscopic examination of the affected cells and tissues [4]. Because there is a dearth of resources for preventing and treating oral cancer, the disorders have 50% to 60% of the total lesions in the oral cavity [5]. Over the last three decades, the five-year survival rate has improved, although it is only about 53% to 60% now [3]. Most cases of oral cancer are not detected until they have progressed to an advanced stage, which significantly contributes to the fact that the survival rate has improved only modestly over time [6]. The typical model includes an oral examination and has been used to screen patients for oral disease and other precancerous lesions. Identifying and presenting hereditary diseases and cancers increasingly depend on investigating unique genetic groups that determine the pathologic processes in such diseases. Understanding the standards underpinning the procedures and the entire natural cycles and subatomic designs being evaluated is essential to the feasible planning and translation of these intelligent symptomatic techniques, just as it is with more seasoned, more renowned suggestive ways.

DNA and RNA are found in the nucleus of every cell in the body. Most DNA used in medical applications comes from blood, bone marrow, and tissue samples acquired by biopsy or surgical resection. It is also possible to utilize a buccal scrape. DNA may also be extracted from tissues that have been formalin-fixed and paraffin-embedded. This is quite helpful when analyzing samples from historical records that have been kept for a long time. Nucleic acids can only be recovered from tissues once the protein cross-links introduced by formaldehyde fixation have been broken down by deparaffinization and treatment with proteinase. If the tissue has been stored in formalin for too long or is too old, the preparation will be less effective [6]. PCR may be used to amplify DNA that has been degraded after being collected from fixed tissues. The success rate of RNA isolation from formalin-fixed tissues has been reported to be lower than that from fresh tissues [6,7].

In the laboratory, oral cancer may be diagnosed using antigens and antibodies via immunohistochemistry and other cutting-edge molecular approaches. Not seeing a doctor soon away is seen as the *first loss of time* due to the patient's lack of awareness, the denial phase, and disregarding the symptoms. The *second loss of time* results from medical and dental practitioners' ignorance and a delayed diagnosis. The interval between the diagnosis and the start of therapy is known as the *third loss of time* [8]. By raising self-examination awareness and educating medical and dental workers, we may decrease the *second loss of time* and minimize the *first loss of time*. Most oral cancers begin as potentially carcinogenic tumors (precancerous). Compared to healthy mucosa, potentially malignant lesions (PMLs) are lesions of the oral mucosa that have a higher chance of developing into cancer. Leukoplakia, erythroplakia, oral lichen planus, and actinic cheilitis are the most prevalent precancerous lesions [8]. Patients with oral cancer have a greater chance of survival if their cancer is diagnosed and treated early. The state-of-the-art diagnostic adjuncts available today are a valuable tool for doing just that [7].

Vital tissue staining

Dye containing toluidine blue (TB) binds strongly to precancerous lesions. Lugol's iodine, in conjunction with TB, is effective in diagnosing mouth malignancies and other abnormalities [9]. Nucleic acids like DNA and RNA may be stained with TB, an (acidophilic) metachromatic dye. The mitochondrial DNA in a cell is stained, and cells with an excessive or deficient amount of DNA may be seen in dysplastic and malignant tissue. When the iodine in Lugol's solution interacts with the glycogen, a brown-black stain is produced, allowing the malignant alteration to be clearly defined [9]. TB and Lugol's iodine are helpful in the diagnosis of high-risk individuals and the selection of the biopsy location for the treatment of wide-field malignancies [9]. TB is a proper, quick, affordable, and efficient supplementary diagnostic technique for locating several PMLs. TB has been shown to help choose biopsy locations and define lesion borders. Cancer has been associated with loss of heterozygosity (LOH) at 3p and 17p, whereas LOH at 9p has been connected to dysplasia, as indicated by recent studies of TB-stained lesions [10]. The methylene blue staining method employs a dye of 1% methylene blue, 1% malachite, 0.5% eosin, glycerol, and dimethyl sulfoxide. Methylene exhibits proportional binding to DNA nucleotides, which explains a rise in stain color intensity with an increase in chromatin material in possibly malignant cells [11].

Vizilite chemiluminescence

The Food and Drug Administration (FDA) has blessed this product to be sold and used in the United States (November 2001). It has been studied and shown that Vizilite improves traditional visual assessment. An individual-use, disposable chemiluminescent light stick produces light at 430, 540, and 580 nm. This may be handled with one hand. Hyperkeratinized or dysplastic lesions reflect light and seem white, whereas normal epithelium (EP) absorbs light and appears black [12]. Use a rinse of diluted acetic acid to check for any changes and monitor them using a chemiluminescent light, such as Vizilite, to detect oral cancer early. Recent studies on high-risk people have demonstrated that Vizilite with TB may accurately identify lesions with a histological diagnosis of dysplasia or cancer in situ. Soft tissue issues may be discovered with technology like Vizilite Plus, which flashes light inside the mouth. This draws attention to the abnormal tissue by demonstrating that it shines in a manner that is different from that of normal tissue. The method has the potential to save lives and is noninvasive and efficient. In any case, it cannot conclusively determine if they constitute a cancer risk [11].

Brush cytology

Since its inception in 1999, brush cytology (Oral CDX, CDx Diagnostics, Suffern, NY, USA) has gained widespread use in modern dentistry clinics. An adjuvant approach has dramatically aided the early diagnosis of oral premalignant and malignant lesions (OPMLs) in recent decades. In this light, Oral CDx may help evaluate dysplastic alterations in a wide range of suspicious lesions, most notably oral cancer [13]. Brush cytology is a low-cost, no-pain, risk-free option. A cytobrush (a thenon laceration device) is used to collect cells from the oral EP, which are then placed on a glass slide, stained with a modified Papanicolaou test, and evaluated using a cutting-edge microscope setup. However, brush cytology has been linked to rumors and false-negative results regarding identifying tumors’ oral epithelial dysplasia. As squamous cell carcinomas (SCCs) account for most oral cancers, this approach has been viewed with skepticism. It is associated with a greater frequency of false-positive side effects than others. It has been seen that additional cutting-edge diagnostic tools are still required [11].

VELscope

The Federation Dental Association has certified VELscope (LED Dental, Inc., Vancouver, BC, Canada), a portable optical instrument, to observe autofluorescence in the mouth directly. It has just been available to medical professionals as a useful diagnostic tool for spotting the first signs of oral illness. It has been helpful for screening for and limiting the spread of many types of cancer, including lung, cervix, skin, and oral cavity malignancies. The primary rationale for using tissue autofluorescence in identifying dysplastic lesions in the oral cavity hinges on changes that light alters the architecture and absorption of the EP and the subepithelial stroma [14]. Tissue healing mechanisms, such as the disintegration of collagen grids and elastin sheets, are assumed to be responsible for the absence of autofluorescence in dysplastic and damaged tissue. The normal mucosa of the body fluoresces a bluish-green. It helps doctors see cancerous lesions that might otherwise go undetected in bright light and better distinguish between benign and malignant alterations. However, this device was unable to discriminate high-risk from low-risk lesions. Thus, it is unreliable for screening PMLs and oral cancer [15].

Confocal microscopy

This imaging method offers the benefits of optical sectioning and high-resolution imaging to a wide variety of cell biology studies. This method helps distinguish oral SCC (OSCC) from normal oral mucosa by detecting markers such as nuclear abnormality. Nonetheless, the instrument has to be fine-tuned even further. Confocal pictures captured in the mouth while the patient was still alive reveal the telltale signs of OSCC, such as nuclear irregularity, that set it apart from healthy oral mucosa. A more refined version of the device is still required before it can be considered a reliable noninvasive tool for early oral cancer diagnosis [16].

Saliva-based oral cancer diagnostics

Saliva is a relatively new idea for detecting OSCC. Saliva or oral fluid is a readily available, minimally intrusive, and very effective diagnostic medium. Diagnostics based on a patient's saliva transcriptome may one day be used to diagnose oral cancer [17]. Patients with head and neck conditions had abnormally high levels of Capnocytophaga gingivalis, Prevotella melaninogenica, and Streptococcus mitis in their saliva, in addition to abnormally high levels of tumor suppressor gene (TSG) *p16*, O6-methyl guanine-DNA methyltransferase, and death-related protein kinase. Nonetheless, evidence is still lacking to support the claim that sound might be a valid diagnostic sign [18]. Although there is growing interest in the study of saliva testing for genetic patterns associated with oral cancer, this technology has not yet been implemented into a commercial product. However, researchers are optimistic that it will soon be widely accessible on the market. Failure to diagnose oral cancer in its early stages significantly contributes to its high fatality rate. It has been shown that using saliva to identify oral cancer is a historical aim that must be achieved for people in the future for better and quicker treatment of the disease at all stages [8].

DNA ploidy and quantification of nuclear DNA content

DNA ploidy, or the number of copies of DNA in a cell's nucleus, may be used as a proxy for the severity of genetic damage. In many cancers, aneuploidy arises because of uneven chromosome distribution between daughter cells during mitosis or because of the separation of chromosomal segments. Cancer detection with DNA image cytometry is susceptible and requires no tissue sampling [19]. Oral epithelial neoplasia, and hence oral cancer, may be detected early using a noninvasive and susceptible technique - cytology with DNA cytometry [18].

Tumor markers and biomarkers

Tumor markers exist in different cell types' plasma, bodily fluids, cell membranes, and cytoplasm. When released by cancer cells or produced by the host in response to cancerous substances. Three of the most reliable indicators of OSCC progression include inherited mutations in the TSG *p53*, chromosomal polysomy (DNA ploidy), and abnormalities (called LOH) in chromosomes 3p or 9p (likely because of changes in TSG *p16*) [19]. Analytical tests analyzing cancer silencer characteristics, oncogenes, cell expansion markers, angiogenic pointers, and cell grip atoms may be used to understand the prognosis of people with OSCC. The research found that cytokeratin markers may also be utilized to identify OSCC by examining the changed keratin expression in oral sites, particularly the buccal mucosa. Exfoliated cells have undergone the same modifications in molecular analysis as tumor biopsy specimens [20].

Diagnostic aids based on PCR

PCR, a method from molecular biology, may be used to identify infectious diseases and investigate tumors that microbes may cause. Cancer research is aided by PCR, which helps shed light on the complex pathophysiology of neoplasia. PCR is a valuable detection method as it can identify both oncogenes linked to cancer (such as *K-ras* and *Nras*) and tumor suppressor genes (like *p53* and *p16*) [21]. Although PCR technology has broadened the breadth and sensitivity of diagnostic methods, contamination and amplification artifacts may raise issues in interpreting the intended results [21]. The PCR method has broadened the applicability and accuracy of diagnostic testing. The significant problem is that it is still susceptible to contamination and amplification artifacts, both of which may make it more challenging to interpret data. While this approach has great potential for improving diagnostic accuracy, it is not yet extensively employed because of its high cost, as some studies have shown [20].

Autofluorescence spectroscopy

One method that has shown promise for finding oral cancer is autofluorescence spectroscopy. The instrument is made up of an optical fiber of varying diameters that produces a spectrum of excitation wavelengths and a spectrograph that records the spectra of fluorescence reflected from the tissue for subsequent computer analysis [22]. However, the method is divisive and often yields ambiguous findings. It is a noninvasive tool for finding signs of sick tissue by analyzing cellular changes in structure and chemistry. It may help the doctor choose the best site for a biopsy. It has been shown via research that a camera-based autofluorescence photodetection technology employing violet excitation light may be a handy diagnostic tool for oral cancers [23].

Fluorescence photography

Fluorescence photography is an effective tool for identifying oral cancer as it is noninvasive, rapid, simple, and reproducible. Carcinoma growth and disease progression could be seen by positive fluorescence staining. The technique is often used in SCC diagnosis. One study found that fluorescence photography was an effective method for detecting oral cancer, particularly SCC [24].

Optical coherence tomography

Initially described by Bandeira et al. [25], it has since found use in several fields, including dentistry, ophthalmology, dermatology, and gastrointestinal. Optical coherence tomography (OCT) is an interferometer-based, noninvasive optical diagnostic instrument [2]. There is hardly a ton of indicators for oral cancer detection, but the EP layer thickness and the standard deviation (SD) of OCT signal intensity are two. The dysplastic cells of an abnormal oral EP are more variable in size, shape, nucleus size, and organization than those of a normal oral EP. The intensity of light scattering and the degree of spatial distribution fluctuation increase in this situation. The patient is not subjected to ionizing radiation or invasive procedures using OCT to produce cross-sectional pictures of normal or diseased tissues [26].

In situ hybridization

Histological and cytological gene expression measurements may be combined with molecular biology methods to create in situ hybridization (ISH). This way, RNA and DNA may be pinpointed to individual cells with ease. A scientific method that has had recent use in diagnostic pathology and microbiology for tissue localization of infectious pathogens [23]. Using the tremendous amplification capability of PCR, in situ PCR, and in situ reverse transcription (RT)-PCR has recently been developed as a further application of ISH, allowing for the detection of meager amounts of nucleic acids in tissues [27].

Identafi 3000

This method utilizes fluorescence, fiber optics, and confocal microscopy in tandem with conventional anatomical imaging to accurately map and outline the lesion in the screened region (Identafi 3000, DentalEZ, Malvern, PA, USA). It is compact, so reaching all of the tissues in your mouth is simple. In a manner analogous to a VELscope, changes in angiogenesis may be seen by shining a special green-amber light on the tissue [22]. 

Microarray

The development of tissue microarrays has made it possible for experts to compare the soundness and toxicity of various tissues by analyzing their articulation profiles. Switch recording uses RNA isolated from both cancerous tumors and healthy tissue to produce cDNA. Fluorescent dyes used in RT allow us to distinguish between expression and control cDNA by their fluorescence emission spectra. After the cDNAs are combined, they are used to hybridize the microarray. As the level of articulation of quality rises in the cancer test, the corresponding area on display becomes more strongly linked to the cDNA region that names the growth [2]. DNA microarrays are now being used for the recognition of single-nucleotide polymorphisms (SNPs) in the human genome (Hap Guide Task), variations in methylation patterns, variations in quality duplicate number, elective RNA joining, and identification of microorganisms. Intriguingly, Jeng et al. extended this introduction to odontogenic improvements by comparing three instances of ameloblastoma with three cases of potentially harmful modifications in the teeth (*one ameloblastic carcinoma, one clear cell odontogenic advancement, and one granular cell odontogenic illness*) [28]. Research showed that all six odontogenic cancers had overexpression of genes and proteins involved in intercellular security and receptors utilized by cells to connect to the extracellular grid, such as integrins (alpha 3, 5, 6, 11; beta two and integrin-related PTK2) [28].

Extracellular grids were affected by proteoglycans and type V collagen, a crucial component in forming tissue-specific networks. Comparing the articulation profiles of three malignant odontogenic tumors to those of three benign ameloblastomas reveals down-regulation of the connexins CX26, CX32, and CX43 in the former. These connexins are responsible for forming hole junctions between epithelia, allowing for long-distance cell movement [29,30]. In addition to its involvement in cell division, coronin is increased in malignant biopsies because it is needed for assembling the normal actin cytoskeleton. MYD88, a member of the death receptor family, is overexpressed in malignant tissue and is involved in many detrimental processes, such as faulty development control, cell cycle arrest, and apoptosis [24]. The transcriptional repressor C-terminal-binding protein (CtBP2) acts as a development silencer and plays a major role in oncogenesis, together with the Ser/Thr nuclear protein kinase STK19 and the record co-activator ABT1, all linked to suppressing highlights in malignant tumors [20]. As with many other characteristics, the DNA-limitation protein, i.e., red fluorescent protein (RFP) has been muted [25].

The future of diagnostic techniques

Chip Laboratory

Microfluidics, micro-total-analysis systems (TAS), or lab-on-a-chip process involves incorporating the steps typically performed in an analytical laboratory onto a single device, or *chip*, and then automating and miniaturizing the whole thing. Many agree that microfluidics will impact other fields, similar to the integrated silicon chip on electronics, computers, and communications [14]. Living cells (typically a few micrometers in diameter) may be easily handled in a microfluidic device because of the three-dimensional, physiologically realistic environment it provides. This microfluidic chip is saliva-friendly, requires basic operator training, and returns an immediate, accurate diagnosis. Oral precancerous (dysplastic) and cancer cells may be identified with this chip by their expression of membrane-associated cell proteins [31].

Nuclear Magnetic Resonance Microscopy

Through this cutting-edge diagnostic tool, a pathologist may investigate the tissue's cellular architecture, examine individual cells for mutations in the genes that regulate their development and function, and identify disease-related biomarkers. This will make it possible to see individual cells in live tissue in three dimensions without harming them. Metabolic imaging at the cellular level using cytomere will be a breakthrough technology [28].

Clinical Microbiology

Microbial research and infectious diseases will benefit significantly from lab-on-a-chip developments such as automated DNA, RNA, and protein/peptide extraction chips coupled with creature ID chips and sequencing chips, providing continuous investigation of patient samples [29].

Cytogenetics

New disease loci are mapped and sequenced using interphase cytogenetic fine-gene mapping, and patients with these mutations are then followed over time [16].

Application of cytogenetics in oral pathology: The t(11;22) (q24;q12) chromosomal translocation repeatedly occurs in around 85% to 90% of instances within the Ewing family of malignancies. More than half of cases have secondary chromosomal abnormalities, most notably gains on chromosome arms 1q, 8, and 12. Sequencing of the resultant fusion protein confirmed that the 5' end of the Ewing's sarcoma (EWS) gene on 22q12 had joined with the 3' end of the *FL1* gene on 11q24, revealing the presence of the t(11;22) breakpoints. Both genes are members of the ETS transcription factor family [26]. SCCs account for 95% or more of all upper aerodigestive tract cancers. Short-term culture has been linked to clonal aberrations in around 200 recorded instances [32,33]. These cases had somewhat complex karyotypes, with many numerical and asymmetrical structural rearrangements. When doing a banding analysis, the most frequent abnormalities include a loss of 2q34-qter, 3p, 4p, 4q28-qter, 8p, 9p13-pter, 10p, chromosomes 13 and 14, 15p, 17q23-qter, 18q21-qter, 21p, 22p, and the Y chromosome, and a gain of 3q, chromosome 7, 8q, and 11q13. Gains and losses have been distributed similarly, but with somewhat varied frequencies, in the few examples evaluated by the Center for Global Health (CGH) thus far [34,35]. Adenocarcinoma, acinic cell carcinoma, mucoepidermoid carcinoma, adenoid cystic carcinoma, and carcinoma ex pleomorphic adenoma are less well defined than SCCs of the head and neck area as there are fewer than 25 relevant cases per tumor type. These tumors frequently have simpler karyotypes than those seen in SCC. Common chromosomal abnormalities in adenocarcinomas and acinic cell carcinomas include 6q deletions and 7p or 8p trisomies. In mucoepidermoid carcinomas, a recurrent t(11;19) (q14-21; p12) has been described; in adenoid cystic carcinomas, a recurrent t(6;9) (q21-24; p22-24) or del(6q) has been described; malignant transformation is frequently accompanied by cytogenetically detectable clonal evolution; and carcinomas arising from pleomorphic. Osteosarcomas have clonal chromosomal abnormalities almost universally. Errors are multifaceted, including several numerical and structural changes. The most frequent imbalances are +1, -2, and -3, and they often occur in the chromosomal regions 1p11-13, 1q11-12, 1q21-22, 11p14-15, 14p11-13, 15p11-13, 17p, and 19q13, -6q, -9, -10, -13, and -17 [36].

Other advanced oral diagnostic aids include a multispectral digital microscope; time-resolved, laser-induced fluorescence spectroscopy; spectroscopy of diffuse reflectance; terahertz imaging; hyperspectral imaging; confocal laser endomicroscopy; quantum dots and nanoparticles; bionic sensor; and diagnostic molecular pathology. The circulating tumor cells (CTCs) assay is a blood test that counts epithelial tumor cells and compares the results to predetermined frequency patterns [37]. The time it takes for radiographic imaging to forecast a patient's response to treatment might be far less than the current clinical practice of two to three months. The FDA has permitted this gadget to be used as a prognostic and monitoring tool for patients with advanced breast cancer [38].

A Dried Blood Spot

A dried blood spot (DBS) is used as the specimen for the test. The process involves breaking down proteins into smaller building blocks called peptides, which are subsequently measured by a mass spectrometer for diagnostic purposes. Taking a blood sample is easy and quick. DBS specimens may be used in genetic testing and molecular diagnostic assays [39,40]. As an alternative to a traditional optical screening colonoscopy, CT scan technology allows for the performance of a virtual colonoscopy. Using the CT scan, Volumetric cine (VC) creates two- and three-dimensional images of the intestinal luminal surfaces [41].

## Conclusions

The key to preventing oral cancer from progressing to later stages is dentists' expertise and training in diagnosing the disease at its precancerous stage. Forecasts for the future of histopathology indicate that molecular pathology will advance in tandem with diagnostic macroscopic and microscopic techniques. Oral malignant lesions have become common in the clinical scenario for which pathology may play an essential role in diagnosis. Early diagnosis can also prevent the lesions from turning into malignancy, leading to the development of individual-specific care at early stages. The significance of these cutting-edge, future-looking diagnostic clinical approaches becomes more apparent when one considers the critical need for early detection and prompt treatment of PMLs and OSCCs. Patient care and diagnostic tool accuracy can be improved to improve early lesion diagnosis and the general population's health and quality of life.
